# Minimum Data Set for a Poisoning Registry: A Systematic Review

**DOI:** 10.22037/ijpr.2020.113869.14538

**Published:** 2021

**Authors:** Azam Sabahi, Farkhondeh Asadi, Shahin Shadnia, Reza Rabiei, Azamossadat Hosseini

**Affiliations:** a *Department of Health Information Technology and Management, School of Allied Medical Sciences, Shahid Beheshti University of Medical Sciences, Tehran, Iran. *; b *Ferdows* *Chamran Hospital, Birjand University of Medical Sciences, South Khorasan, Iran. *; c *Toxicological Research Center, Department of Clinical Toxicology, Loghman Hakim Hospital, School of Medicine, Shahid Beheshti University of Medical Sciences, Tehran, Iran.*

**Keywords:** Minimum data set, Data set, Registry, Data management, Poison, Toxic

## Abstract

Poisoning, as a well-known medical condition, puts everyone at risk. As a data management tool, a registry plays an important role in monitoring the poisoned patients. Having a poisoning minimum data set is a major requirement for creating a poisoning registry. Therefore, the present systematic review was conducted in 2019 to identify the minimum data set for a poisoning registry. Searches were performed in four scientific databases, *i.e*., PubMed, Scopus, Web of Science, and Embase. The keywords used in the searches included minimum data set, “poison”, and “registry”. Two researchers independently evaluated the titles, abstracts, and texts of the papers. The data were collected from the related papers. Ultimately, the minimum data set was identified for the poisoning registry. Data elements extracted from the sources were classified into two general categories: administrative data and clinical data. Ninety-eight data elements in the administrative data category were subdivided into three sections: general data, admission data, and discharge data. One-hundred and thirty-one data elements in the clinical data category were subdivided into five sections: clinical observation data, clinical assessment data, past medical history data, diagnosis data, and treatment plan data. The minimum data set is a prerequisite for creating and using a poisoning registry and data system. It is suggested to evaluate and use the poisoning minimum data set in accordance with the national laws, needs, and standards based on the opinion of the local experts.

## Introduction

Poisoning is defined as the emergence of symptoms in an organism due to exposure to harmful chemical, physical, or organic substances (1). It is accompanied by a manifestation of the symptoms depending on the type and amount of the toxin, exposure time, and means of contact with or entrance into the body (2). Acute poisoning, as a well-known medical condition, puts everyone at risk. Children, teenagers, women of reproductive age, and the elderly are more likely to be at risk than the others (3, 4). Studies have shown that acute poisoning is a very common condition in people referring the emergency rooms across the world, requiring costly medical attention (5-8). In addition, this type of poisoning is constantly on the rise due to the changes in lifestyles and social-psychological pressure. Advancements in the technology and growth of societies have provided easy access to pharmaceuticals and chemical substances in many countries, thus leading to acute poisonings (9). 

The world health organization (WHO) has estimated that the number of cases of acute unintentional poisoning ranges between 3.5-5 million cases around the world each year. Among these, 3 million cases are severe, leading to 20,000 deaths annually (10). The most common cases of intentional poisoning in industrial countries involve overdoses by painkillers, anti-anxiety medications, and anti-depressants. However, the most common causes in the Asian countries are agricultural pesticides resulting from self-poisoning, especially in the rural areas, where it has a 10-20% lethality rate (11, 12). Different factors contribute to the rise in the number of poisoning cases in developing countries, including insufficient laws on the use of pharmaceuticals and medicinal chemicals, weak regulatory systems, and easy access to various pharmaceuticals and medicinal chemicals (13). The consequences of poisoning in these countries can be much more serious than the high-income countries (14). 

Data collected by the WHO in 2012 revealed that 193,460 people die from unintentional poisoning each year, and 84% of these cases occur in medium- and low-income countries (15). It has also been shown that nearly 1 million people die because of suicide every year, and the use of chemicals is the cause of a considerable percentage of them (16). 

Studies have found that the rate of mortality resulting from the poisoning is not the same for all societies, but it depends on the societal and population factors, such as ethnicity, age, sex, geographical location, and the level of economic development (17-19). Therefore, knowledge of the general patterns of poisoning in different geographical regions can help in prevention, early detection, identification of the risk factors, and management of the poisoning cases, thereby reducing the rates of disease and death (20). 

Implementing a poisoning prevention and management program through the use of data organization and management will be a big step towards improving the quality of care, disease control, and promoting the health of the society as a whole (21). Many countries possess specialized databases for managing the medical products and substances available in their region. Therefore, a database of toxins-related information will play a determining role in the prevention and management of drug poisoning cases by providing the information to the specialists and the general public (22). A registry is an effective information system with the ability to create databases and, therefore, can provide an important source of information about the medical patterns, decisions, and treatments for the healthcare providers and researchers, and also assist them in discerning the links between the causes and outcomes of diseases (23-26). 

A poisoning registry database improves the quality of clinical care. This approach also helps to disseminate the information regarding antidotes or treatment methods, newer management options and identify the risk factors in managing the poisoned patients. This database is also expected to assist in identifying the regions and societies potentially at risk of poisoning. Implementing the registry database will help the different sectors access advanced educational instructions regarding the harmful effects of the toxins (27). Registry databases usually employ the minimum data sets (MDSs) to facilitate the precise analysis of the data, decision-making, and correct management of the poisoning cases (28, 29). 

The MDS is a common collection of the data that must be used for gathering and reporting the data in a registry (30). An MDS is an effective tool in data collection, providing accurate access to medical information systems. It is extremely useful for planning, developing, supervising, managing, and assessing the performances, improving the quality of care and disease control, and reducing the costs (31, 32). In addition, MDSs provide the standard data, which can be used for external validation, internal performance assessment, and national and international comparisons (33). The creation of a national database is the main aim of an MDS. An MDS can be utilized as an information management source to equip the policy-makers and decision-makers with accurate and up-to-date information (34-36). The WHO also stresses that the major goal of an MDS is supporting mutual planning in different countries (37). 

Therefore, an MDS appears to be critical for creating a database used for the collection, processing, and dissemination of the information (38). To this end, the American college of medical toxicology (ACMT) founded the toxicology investigators consortium (ToxIC) (39). An Internet-based poisoning database named TOXBASE was established in England in 1999 (40). Australia has the hunter area toxicology service (HATS) offering these services (41). Moreover, Korea has created a web-based poisoning information database (PIDB) to deliver information on the emergency medical care of the poisoned patients (42). There has been no study on the MDS for a poisoning registry to the best of our knowledge. Since the poisoning MDS is necessary for continuous collection and storage of the data and is a major prerequisite for creating and using of a registry and information system. The present systematic review was conducted to identify the MDS for a poisoning registry.

## Experimental

*Information Sources and Search Strategy*

The present systematic review was done to identify the MDS for a poisoning registry. The search strategy was designed for each database with inputs from the authors and based on the previous studies by combining three groups of keywords related to the subject. The included keywords described an MDS, registry, and poisoning. Then, the searches were performed in each of the following databases: PubMed, Scopus, Web of Science, and Embase, for the entries registered until May 8^th^, 2019 (using the medical subject headings [MeSH], truncation symbols, and Boolean operators). Table 1 presents the keywords used to search for the related papers.

*Eligibility Criteria*

The present systematic review was conducted based on the PRISMA (preferred reporting items for systematic reviews and meta-analyses) (43). 

*Inclusion Criteria*

No language limitation was set for including the resources. Papers mentioned the human poisoning data elements in poisoning or related databases were included in the review.

*Exclusion Criteria*

 Papers with non-sufficient details about the poisoning data elements were excluded from the study. Papers other than the original research papers (*e.g*., protocols, editorials, and reviews) were also excluded, but their reference lists were checked. Papers whose full text could not be accessed for any reason were also excluded. Moreover, papers that had presented the data elements from the poisoning databases of a particular country but at different time points were excluded. Only one with a complete report of the data elements regarding the poisoning database of that country was included.

*Study Selection*

Two researchers independently evaluated the titles and abstracts of all the retrieved papers during the screening stage and excluded the irrelevant ones based on the inclusion and exclusion criteria. In the eligibility stage, two researchers independently studied the texts of all the papers not excluded in the previous stage and selected those meeting the inclusion criteria. Disputed cases were resolved by a third independent researcher. The reference lists of the included papers were screened for other ones that could meet the inclusion criteria. 

Hand searching was also performed in the Journal of Clinical Toxicology and Google Scholar database. The most prominent authors were contacted and requested gray literature, including the conference papers with an available full text, reports, and unpublished research.

*Data Collection Process*

One reviewer extracted the data from the included papers, and the second reviewer evaluated the extracted data. Cases of disagreement were resolved through discussion between the two reviewers. The data were extracted using a structured table based on the following parameters: the first author’s last name, the year of publication, the country where the study was conducted, and data elements. 

Data elements were sorted into two main categories: administrative data and clinical data. This kind of categorization is employed in the widely accepted sources of healthcare data classification (29, 44, 45). 

*Assessment of the Methodology Quality*

Methodological quality was assessed independently by two reviewers through the standardized tools for critical appraisal based on strengthening the reporting of observational studies in epidemiology (STROBE) guidelines (46). The STROBE checklist was selected, as the included studies were observational.

 The quality score was classified into six sections, and three categories from Category A to Category C. Table 2 presents the details for the STROBE checklist.

Discussions were held with a third independent researcher to resolve the disagreements between the reviewers. 

## Results

*Summary of the Study Characteristics*

*Number of Studies*

**Figure 1:**
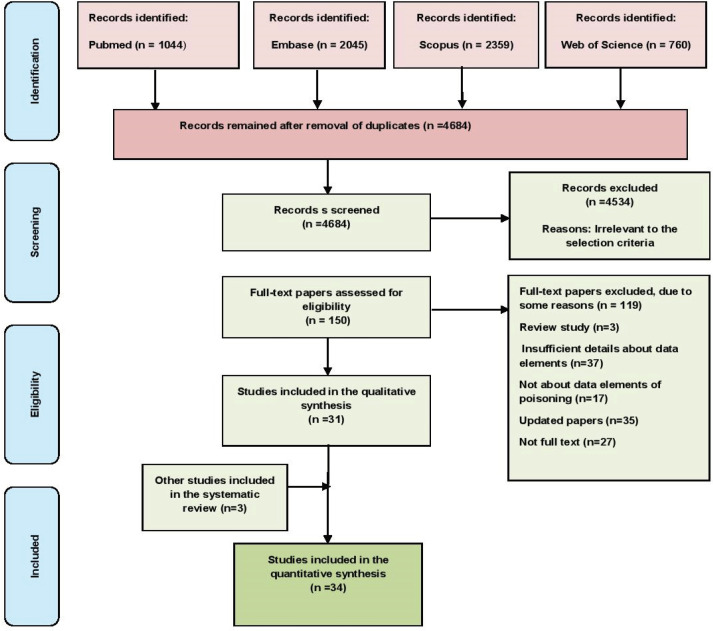
Flow diagram of the included and excluded studies

A total of 6208 papers were retrieved in an initial search in the four databases, and they were subsequently imported into the reference management software, EndNote. After removing the duplicates and unrelated papers based on their titles, abstracts, and texts, 34 papers were ultimately chosen. Figure 1 shows the workflow of the paper selection.

*Sources of the Studies*

Most of the studies had been conducted in the US (47-63), and the others had been carried out in Turkey (64), Japan (65), Australia (41, 66), Poland (67), Spain (68), Italy (69, 70), Zimbabwe (71), Oman (72), Saudi Arabia (73), Pakistan (74), Korea (75), France (76), Israel (77), Iran (45), and Hong Kong (78). 

*Quality Assessment*

The quality of most of the papers was good (41, 45, 47-56, 58-66, 68 and 70-78), and it was moderate in three cases (57, 67, 69). The quality score was obtained as 19 in six studies (45, 51, 54, 56, 60 and 71), 18 in 14 studies (41, 48-50, 58, 59, 61, 62, 64 and 72-76), 17 in six studies (47, 53, 66, 70, 77 and 78), 16 in four studies (52, 63, 65 and 68), 15 in one study (55), and 14 in three studies (57, 67 and 69).

*Classification of the Data Elements*

Data elements were extracted from the studies and were sorted into two general categories: administrative data and clinical data. In the administrative data category, 98 data elements were sorted into three sections: general data (n = 24 data elements), admission data (n = 37 data elements), and discharge data (n = 37 data elements). The most prevalent data elements in the general data subcategory were related to age (100%, n = 34 out of 34 studies) and sex (91.17%, n = 31 out of 34 studies). In comparison, the data elements with the highest frequency in the admission data subcategory were related to the location encounter (32.35%, n = 11 out of 34 studies), followed by the patients҆ code and admission time (14.70% for each of them, n = 5 out of 34 studies). The most frequent data elements in the discharge data subcategory were related to the outcome (52.94%, n = 18 out of 34 studies), followed by the length of hospital stay (20.58%, n = 7 out of 34 studies). In addition, 131 data elements in the clinical data category were classified into the following five sections: clinical observation data (n = 24 data elements), clinical assessment data (n = 79 data elements), past medical history data (n = 7 data elements), diagnosis data (n = 2 data elements), and treatment plan data (n=19 data elements). The most frequent data elements in the clinical observation data subcategory were related to the symptoms (61.76%, n = 21 out of 34 studies), followed by the signs (29.41%, n = 10 out of 34 studies). The data elements in the clinical assessment data subcategory were subdivided into the following two sections: exposure data (n = 52 data elements) and paraclinical tests҆ data (n = 27 data elements). Moreover, the most frequent data elements in the exposure data section were related to the reason for encounter (70.58%, n = 24 out of 34 studies), followed by the route of exposure (61.76%, n = 21 out of 34 studies). The most prevalent data elements in the paraclinical tests҆ data section were related to the laboratory tests (20.58%, n = 7 out of 34 studies), followed by the laboratory results (11.76%, n = 4 out of 34 studies). The most frequent data elements in the past medical history subcategory were related to the comorbidity diseases (14.70%, n = 5 out of 34 studies), followed by the history of psychiatric disorders (11.76%, n = 4 out of 34 studies), while the most common data elements in the diagnosis data subcategory were related to the medical and psychiatric diagnosis (2.94% for each of them, n = 1 out of 34 studies). In the treatment plan data subcategory, the most common data elements were related to the type of treatment (58.82%, n = 20 out of 34 studies), followed by the frequency of treatment, duration of treatment, and surgery (2.94% for each of them, n = 1 out of 34 studies). Tables S1 and S2 (in supplementary file) present the data elements. 

Some further details about certain data elements have been provided in other studies. For instance, details regarding the exposure agents in the study by Mowry include non-pharmaceutical substance exposure in 38 groups and pharmaceutical substance exposure in 30 groups (47). Bentur classified the exposure elements. The chemical agents were sorted into 21 groups, pharmaceuticals were divided into 30 groups, biologic agents were classified into nine groups, and miscellaneous agents were sorted into seven groups (77). Meanwhile, some papers have provided more details on the type of treatment (47, 60). Farrugia divided the antidotal therapy into 29 groups, antivenom therapy into five groups, supportive care—pharmacological into 13 groups, supportive care—non-pharmacological into 11 groups, decontamination into four groups, chelation therapy into four groups, and enhanced elimination into six groups (60). 

## Discussion

The risk of poisoning for the general public is increasing every day due to the rise in the amount of chemicals, pharmaceuticals, and natural toxins. It is crucial to create an information management system to completely collect all the related information promptly to identify the populations at risk, design the programs to control, prevent, and assess the diseases; and enhance the quality of the healthcare system for the poisoned patients. 

Creating a poisoning registry using the MDS can help generate higher quality information, which can lead to better clinical decisions. Expanding the MDS of poisoning database can promote the efficiency of the hospitals and clinical centers. Thus, this systematic review was conducted to identify the MDS for a poisoning registry. According to the findings of the present study, 229 data elements were sorted into the two categories of administrative and clinical data. Most of the data elements in the administrative data category were related to age, sex, location encounter, patients҆ code, admission time, outcome, and the length of hospital stay. In the clinical data category, the most prevalent data elements were related to the symptoms, signs, reason for the encounter, the route of exposure, laboratory results, laboratory tests, comorbidity disease, psychiatric disorders, and the type of treatment. 

In many studies, a combination of source examination and experts҆ consensus has been adopted for developing the MDS. For example, Davey *et al*. (2017) identified a minimum list of international primary care optometry metrics. They proposed the patients’ demographic information, outcome, signs, history of the disease, and results of clinical tests as part of an MDS for primary eye care (32).

Emami *et al*. introduced a population-based registry for multiple sclerosis. They used the MDS defined by the Center for Disease Control and Prevention in Iran’s Ministry of Health and Medical Education, which included the demographic and clinical data, the latter consisting of seven subcategories: the age of onset for symptoms, age of diagnosis, relapse date, the current status of the immune system, symptoms and immunological treatment, the use of healthcare services, and disability level (79). 

Abbasi *et al*. conducted a study to develop an MDS for the infertility registry, in which general information, patients҆ history, paraclinical reports, treatment plan, and treatment outcome constituted the MDS for developing an infertility registry in Iran (80).

Kazemi-Arpanahi *et al. *developed an MDS for electrophysiology study of cardiac ablation and for establishing an information management system or clinical registry, administrative data, past medical history, sign and symptoms, physical examinations, laboratory tests, post-procedure complications, and discharge outcomes were confirmed as part of a set of core data elements (81).

Rampisheh *et al*. conducted a study to design an MDS for hospital information systems in Iran. In this study, data elements were classified into administrative and clinical data. Data classes belonging to the administrative data included the demographic, admission, incidence, legal, discharge, financial, personnel identifier, organization identifier, and geographic. The clinical category comprised the following data classes: diagnosis, pre-hospital emergency, hospital emergency, diagnostic\ therapeutic procedure, orders, medical imaging, laboratory, medicine, medical prosthetics, blood products, discharge status, transfer, follow-up, system history and review, nursing, consultation, death, and anesthesia (82). 

Amerai *et al*. conducted a systematic review to create an MDS for mental health. The data elements were classified into two general categories: management data and clinical data. The data elements belonging to the management group included identifying the admission information, demographics/history, and discharge information. Moreover, the data elements of the clinical group were related to the service event data and assessment of the patient (29). 

The use of administrative data is expanding daily by the planners and public health researchers (83). Lucyk *et al*. (2017) pointed out that administrative data are used to monitor the population, geographical variation, the populations҆ health, and healthcare planning (84). Healthcare providers, financers, and policy-makers incorporate the administrative data to conduct the operations, assess the population outcomes, and measure the quality of healthcare, insurance and reimbursement, medical research, outcome evaluation, and administrative reports (82, 85 and 86). Clinical data are collected by the clinical staff and rely upon the diagnosis and treatment processes and are used to assist the research, planning, and making policies regarding the health (80, 87). These data are also essential for high-quality healthcare, improving the healthcare management, reducing the cost of healthcare, management of the populations҆ health, and effective clinical research, as well as meeting the needs of the financers, healthcare administrators, clinical research, and public health (88, 89). 

In developing countries, all the information about the patients is stored in the national MDS, so they are available for auditing, analyzing, and assessing the quality of the data. The ministries of health in the countries, such as the US and Canada, use the information networks to access the national MDS (90). Most of the researches included in this study about poisoning data elements were from the poison control centers in the US, and some details about these data elements were presented completely in the mentioned studies. The American association of poison control centers (AAPCC) maintains and manages the national poison data system (NPDS) is responsible for overseeing its development (91). 

The NPDS is a data warehouse for over 50 poison control centers and was developed in 1983. It is the only real-time poisoning surveillance system in the US (92). The database includes the entries on more than 390,000 pharmaceutical, chemical, and household products and allows them to be identified by their generic and brand names (93). The ACMT has also created an international registry of the poisoned patients named the ToxIC. It was established in 2010 as a tool for clinical toxicology research to develop the collaboration, education, and research among the physicians specializing in the management of human poisoning cases across the globe to improve the care offered to the poisoned patients (94). 

The ToxIC registry is unique in several ways. Since all the data are entered by treating the medical toxicologists the toxicology information is an indicator of the outcome of the professional work performed by the skilled and specialist physicians. A large part of the information in this database cannot be accessed from any other source, including the clinical data and the demographic data (95). This registry presents the pertinent details to provide the clinical toxicologists with the opportunity to identify the patterns of diseases, important toxins, and effective treatments for poisoning in humans. An additional aim of ToxIC is developing the infrastructure for a multidisciplinary research network (96). 

Mandavia *et al*. (2017), in a systematic review entitled “What Are the Essential Features of a Successful Surgical Registry?” demonstrated that the flexible data sets with the ability to evolve could help increase the longevity of the registries. Their findings regarding the measures of a successful registry revealed that a successful registry is one that can be easily accessed and has a high rate of data completion and participation, which can promote the national and international collaborations. Successful registries are useful for their stakeholders and contain the validated information that can be analyzed easily and accurately (97). Other systematic studies on the MDS have found them to be crucial for continuous recording of the data and a major prerequisite for creation and use of the registries and information systems (29, 98 and^ 99^). They are also useful in meeting the needs of their stakeholders. The results of another study showed that a successful data set should be able to take the needs of the registry users into account and strike a balance between collecting the desired data and limiting factors, which can act as opposing forces. It should also be able to minimize any uncertainty about the definition and classification of the variables in the registry system (100).

Even though it is useful and essential to identify and develop the poisoning MDS, and considering the WHO’s report on the importance of access to information for the advancement of the healthcare systems (28), the MDS should be evaluated and used under the national laws, regulations, and standards of each country based on the opinions of its experts to prevent the collection of unnecessary data, which can lead to an excess of data and an increased workload for the healthcare personnel. 

**Table 1:** Search strategies for different databases

Pubmed	( ("Minimum Data Set"[All fields] OR "Dataset" [All fields] OR "Common data elements" [All fields] OR "Data elements" [All fields] OR "Data recording" [All fields] OR "Data utilization" [All fields] OR "Common data" [All fields] OR "Data collection" [All fields] OR "national data set" [All fields] OR "Core data set" [All fields] OR "Dataset"[Mesh terms] OR "Common data elements" [Mesh terms] OR "Data collection" [Mesh terms]) AND ("Register*"[Title/Abstract] OR "Database*"[Mesh terms] OR "Database management system*"[Mesh terms] OR "information system*"[Mesh terms] OR "Data system*" [Mesh terms] OR "Data management" [Title/Abstract] OR "information management" [Mesh terms] OR "surveillance system" [Title/Abstract] OR "Database*"[Title/Abstract] OR "Database management system*"[Title/Abstract] OR "information system*"[Title/Abstract] OR "Data system*"[Title/Abstract] OR "Database management system*"[Title/Abstract])) AND ("Poison*"[Title/Abstract] OR "toxic*"[Title/Abstract] OR "intoxic*"[Title/Abstract] OR "noxious" [Title/Abstract] OR "Poisons" [Mesh terms])
Scopus	( (ALL ("Minimum Data Set") OR ALL ("Dataset") OR ALL ("Common data elements") OR ALL ("Data elements") OR ALL ("Data recording") OR ALL ("Data utilization") OR ALL ("Common data") OR ALL ("Data collection") OR ALL ("national data set") OR ALL ("Core data set")) AND (TITLE-ABS-KEY ("Registr*") OR TITLE-ABS-KEY ("Database*") OR TITLE-ABS-KEY ("Database management system*") OR TITLE-ABS-KEY ("information system*") OR TITLE-ABS-KEY ("Data system*") OR TITLE-ABS-KEY ("Data management") OR TITLE-ABS-KEY ("information management") OR TITLE-ABS-KEY ("surveillance system")) AND (TITLE-ABS-KEY ("Poison*") OR TITLE-ABS-KEY ("toxic*") OR TITLE-ABS-KEY ("intoxic*") OR TITLE-ABS-KEY ("noxious")))
Embase	#1 ‘Minimum Data Set’:ti,ab,kw OR ‘Dataset’:ti,ab,kw OR ‘Common data elements’:ti,ab,kw OR ‘Data elements’:ti,ab,kw OR ‘Data recording’:ti,ab,kw OR ‘Data utilization’:ti,ab,kw OR ‘Common data’:ti,ab,kw OR ‘Data collection’:ti,ab,kw OR ‘national data set’:ti,ab,kw OR ‘Core data set’:ti,ab,kw #2 ‘Dataset’/de OR ‘Common data elements’/de OR ‘Data collection’/de # 3 # 1 OR #2 #4 ‘Register*’:ti,ab,kw OR ‘Database*’:ti,ab,kw OR ‘Database management system*’:ti,ab,kw OR ‘information system*’:ti,ab,kw OR ‘Data system*’:ti,ab,kw OR ‘Data management’:ti,ab,kw OR ‘information management’:ti,ab,kw OR ‘surveillance system’:ti,ab,kw #5 ‘Database’/de OR ‘Database management system*’/de OR ‘information system*’/de OR ‘Data system*’/de # 6 #4 OR #5 #7 ‘Poison*’:ti,ab,kw OR ‘toxic*’:ti,ab,kw OR ‘intoxic*’:ti,ab,kw OR ‘noxious’:ti,ab,kw #8 Poisons/de # 9 #7 OR #8 #3 AND #6 AND #9
ISI	( (TS= (“Minimum Data Set”) OR TS= (“Dataset”) OR TS= (“Common data elements”) OR TS= (“Data elements”) OR TS= (“Data recording”) OR TS= (“Data utilization”) OR TS= (“Common data”) OR TS= (“Data collection”) OR TS= (“national data set”) OR TS= (“Core data set”) OR TI= (“Minimum Data Set”) OR TI= (“Dataset”) OR TI= (“Common data elements”) OR TI= (“Data elements”) OR TI= (“Data recording”) OR TI= (“Data utilization”) OR TI= (“Common data”) OR TI= (“Data collection”) OR TI= (“national data set”) OR TI= (“Core data set”)) AND (TS= (“Registr*”) OR TS= (“Database*”) OR TS= (“Database management system*”) OR TS= (“information system*”) OR TS= (“Data system*”) OR TS= (“Data management”) OR TS= (“information management”) OR TS= (“surveillance system”) OR TI= (“Registr*”) OR TI= (“Database*”) OR TI= (“Database management system*”) OR TI= (“information system*”) OR TI= (“Data system*”) OR TI= (“Data management”) OR TI= (“information management”) OR TI= (“surveillance system”)) AND (TS= (**”**Poison*”) OR TS= (“toxic*”) OR TS= (“intoxic*”) OR TS= (“noxious”) OR TI= (**”**Poison*”) OR TI= (“toxic*”) OR TI= (“intoxic*”) OR TI= (“noxious”)))

**Table 2:** The STROBE checklist items and scores

**Items on checklist**	**Details**	Score*
Title and abstract	Study design, providing the abstract with an informative and balanced summary	1
Introduction	Scientific background and rationale, specific objectives	2
Methods	Key elements of study design, setting, participants, variables, data sources/measurement, bias, study size, quantitative variables, statistical methods	9
Results	Participants, descriptive data, outcome data, main results, other analyses	5
Discussion	Key results, limitations, interpretation, generalizability	4
Other information	Funding	1

*Category A: scores 15-22 (good quality).

Category B: scores 8-14 (moderate quality).

Category C: scores <8 (poor quality).

## Conclusion

Health information is critical for diagnosis, treatment, and control of the diseases; improvement of the healthcare programs; and support of the clinical decisions. Therefore, the design and implementation of an effective information management system are a high priority for the healthcare system of every country. As one of the most important parts of an information management system, the MDS has shown great potential for helping policymakers and healthcare providers to offer high-quality care and control for diseases.

An MDS is essential for continuous collection and storage of the data and is a major prerequisite for creating and using a poisoning information system and registry. It also provides uniform definitions of the concepts and data elements. It covers the full information regarding the poisoned patients, which is required for large-scale clinical and administrative decision-making.

## Supplementary Materials



## Limitations of the Study

Unfortunately, the full text of some papers was not available, and this was considered as a limitation of this study.
