# Supplementary material for: Minimum Data Set for a Poisoning Registry: A Systematic Review
Source: Iran J Pharm Res. 2021 Spring;20(2):473–85. doi: 10.22037/ijpr.2020.113869.14538 (PMC8457722; doi:10.22037/ijpr.2020.113869.14538)
Supplement: Supplementary file 1 [file ijpr-20-473-s001.pdf]

Supplementary Materials for

Minimum Data Set for a Poisoning Registry: A Systematic Review

Azam Sabahi, Farkhondeh Asadi, Shahin Shadnia, Reza Rabiei and Azamossadat Hosseini

*To whom correspondence should be addressed. E-mail: asadifar@sbmu.ac.ir

Volume 20, Issue 2 (Spring 2021)

This PDF file includes:
Tables S1-S2

Table S1. Administrative data elements for the poisoning registry.

**Subcategory**	**Data Elements**	**References No.**
General Data	Name	(41, 45 and 67)
	Surname	(67)
	Place of Birth	(41, 67)
	Marital Status	(41, 45 and 67)
	Occupation	(41, 45, 49 and 67)
	Education Level	(45)
	Patient’s Address	(41, 45 and 67)
	Patient’s Email Address	(41)
	Patient’s Landline Phone Number	(45)
	Patient’s Mobile Phone Number	(45)
	Age	(41, 45 and 47-78)
	Sex	(45, 47-55 and 58-78)
	Pregnancy Status	(47, 60)
	First Trimester	(47)
	Second Trimester	(47)
	Third Trimester	(47)
	Weight	(41, 47 and 61)
	Height	(41, 45)
	Race	(60)
	Ethnicity	(41, 60 and 62)
	Nationality	(73, 74)
	Region	(49, 64 and 75)
	Rural or Urban	(64, 75)
	Metropolis	(75)
Admission Data	Patients’ Code	(41, 45, 53, 61 and 63)
	Type of Insurance	(45)
	Admission Date	(41, 45 and 67)
	Admission Time	(41, 45, 67, 70 and 75)
	Mode of Arrival	(74)
	Ambulance	(74)
	Non-Ambulance	(74)
	Management Site	(62, 55, 48)
	Emergency Department	(68, 51)
	Managed on Site (Non-Healthcare Facility)	(50, 48, 47)
	Managed in the Healthcare Facility	(47, 48)
	Treated, Evaluated, and Released	(48, 47)
	Admitted to the Critical Care Unit	(68, 48, 47)

	Admitted to the Non-Critical Care Unit	(48, 47)
	Admitted to the Psychiatric Facility	(48, 47)
	Patient Lost to Follow-Up	(48, 47)
	Refused Referral	(47)
	Unspecified Level of Care	(48, 47)
	Other/Unknown	(48, 47)
	Source of Referral	(60)
	Emergency Department	(60)
	Outpatient /Clinic/Office Consultation	(60)
	Location Encounter	(47, 51, 55, 57, 61, 62, 65, 66, 70, 77 and 78)
	Healthcare Facility	(47, 51, 77 and 78)
	Residence	(47, 55, 65 and 78)
	School	(47, 77)
	Workplace	(47, 51, 55, 77 and 78)
	Restaurant/Food Services	(47)
	Public Area	(47)
	Outdoor	(65, 77)
	Home	(51, 77)
	Military Base	(77)
	Agricultural Farm	(77)
	Sea	(77)
	Industrial Plant	(77)
	Other	(47, 55, 65, 77 and 78)
	Unknown	(47, 77, 78)
Discharge Data	Date and Time of Discharge	(41)
	Length of Hospital Stay	(45, 54, 57, 64, 68, 71 and 72)
	Healthcare Provider	(41, 45, 54, 56, 70 and 74)
	Location, Name	(45, 54)
	Type of Care Provider	(45, 54, 56 and 74)
	Emergency Medicine	(56)
	Internal Medicine	(56)
	Family Practice	(56)
	General Practice	(56)
	Nurse Practitioner or Physician's Assistant	(56, 74)
	Toxicology, Occupational, Preventive Medicine	(56)
	Paramedic	(56)
	Intern	(56)
	Resident	(56)
	Type of Hospital	(74)

	Public Hospital	(74)
	Private Hospital	(74)
	Types of the Used Services	(56, 74)
	ED (Emergency Department)	(74)
	ICU (Intensive Care Unit)	(74)
	Observation Unit	(74)
	Protective Services	(56)
	Level of ED	(75)
	Level 1	(75)
	Level 2	(75)
	Level 3	(75)
	Outcome	(41, 47, 48, 50, 52, 55, 57, 58, 61-64, 70, 72-75 and 78)
	Death	(47, 48, 55, 58, 64, 68, 70, 72, 73 and 78)
	Death, Indirect Report	(47)
	Residual Consequences	(72)
	Major Effect	(47, 48, 52, 55, 58, 64 and 78)
	Minor Effect	(47, 48, 52, 55, 58, 64 and 78)
	Moderate Effect	(47, 48, 52, 55, 58, 64 and 78)
	Complete Recovery	(72, 73)
	Invalid or Missing	(58)
	Unknown	(47, 52, 55, 58 and 78)
	Follow-Up	(41)

Table S2. Clinical data elements for the poisoning registry

Subcategory	Data Elements	References No.
Clinical Observation Data	**Symptom**	(45, 47, 49, 51-58, 60-65, 67, 68, 70 and 76)
	Pulmonary	(45, 49, 51 and 60)
	Gastrointestinal	(45, 49, 51 and 60)
	Neurological	(45, 49, 51 and 60)
	Ocular	(49, 51)
	Dermal	(45, 49 and 51)
	Cardiovascular	(45, 49 and 60)
	Toxidromes	(50, 60)
	Metabolic	(45)
	Miscellaneous Symptoms	(49)
	**Signs**	(41, 47, 57, 60-62, 68-70 and 76)
	Neurological	(41, 60, 68 and 76)
	Cardiovascular and Pulmonary	(60)
	Metabolic	(60)
	Renal/Musculoskeletal	(60)
	Hematological	(60)
	Gastrointestinal/Hepatic	(60)
	Toxidromes	(41)
	**Vital Signs**	(41, 45, 50, 54 and 60)
	Level of Consciousness (Including Glasgow Coma Score)	(41)
	Blood Pressure	(41, 50 and 60)
	Respiratory Rate	(41, 60)
	Pulse	(41, 50 and 60)
	Temperature	(41, 50, 60)
Clinical Assessment Data	**Exposure Data**	
	**Type of Encounter**	(56, 72)
	Acute	(72)
	Chronic	(72)
	**Reason for Encounter**	(47, 48, 50, 52-58, 60, 61, 63-65, 68, 70-74 and 76-78)
	Unintentional/Accidental	(47, 48, 50, 52, 55, 60, 64, 65, 71-73 and 76-78)
	Intentional	(47, 48, 52, 55, 60, 71-73 and 76-78)
	Suicide	(48, 50)

	Recreational	(50)
	Adverse Reaction	(47, 52, 76 and 78)
	Unknown	(47, 48, 50, 52, 60, 64, 71, 73, 76 and 78)
	Other	(47, 50, 55, 65, 72 and 78)
	Not Recorded	(60)
	**Duration of Exposure**	(53, 61, 62 and 69)
	**Severity of Exposure**	(49, 51, 61, 64, 76 and 77)
	No Effect	(51, 77)
	Minor	(49, 51, 64, 76 and 77)
	Moderate	(49, 64, 76 and 77)
	Major	(49, 51, 64, 76 and 77)
	Unknown	(77)
	**Date and Time of Exposure**	(41, 53, 55, 67, 70 and 72)
	**Activity During Exposure**	(66)
	**Route of Exposure**	(41, 47, 50-53, 55-59, 62, 64, 65, 69-73, 77 and 78)
	Ingestion	(47, 50-52, 56, 58, 65, 72, 73 and 77)
	Transdermal	(47, 51, 56, 58, 59, 65, 72, 77 and 78)
	Inhalation/Nasal	(47, 50, 51, 56, 58, 59, 65, 72, 73, 77 and 78)
	Ocular	(47, 51, 56, 58, 59, 65, 72, 77 and 78)
	Parenteral	(47, 52, 59 and 78)
	Otic	(47)
	Aspiration	(47)
	Vaginal	(47)
	Rectal	(47, 77)
	Bite/Sting	(47, 51, 58, 65, 72 and 77)
	Injection	(50, 72)
	Multiple	(58)
	Insufflation	(50)
	Other	(47, 50, 51, 58, 59, 73, 77 and 78)
	Unknown	(47, 50-52, 58 and 77)
	**Exposure Dose and Frequency**	(54, 59, 69, 70, 72 and 76)
	Mg	(59)
	G; Mg/Kg; Ml/Kg	(59)
	Other	(59)
	**Risk Assessment of Poisoning**	(54, 72)
	Nontoxic	(72)
	Mild	(72)

	Moderate	(72)
	Severe	(72)
	Critical	(72)
	**Exposure Agent**	(41, 45, 47-49, 51, 53, 57, 58, 60, 70-73, 75, 77 and 78)
	Non-Pharmaceuticals /Chemicals	(47-49, 51, 53, 57, 58, 60, 70-73, 75, 77 and 78)
	Pharmaceuticals	(47, 48, 51, 53, 57, 58, 60, 66, 70-72, 75, 77 and 78)
	Biologic Agents	(77)
	Miscellaneous Agents	(77)
	**Paraclinical Tests̉ Data**	
	**Laboratory Tests**	(41, 45, 47, 50, 54, 67 and 71)
	ABG (Arterial Blood Gas)	(45)
	WBC (White Blood Cell) Count	(45)
	Hb (Hemoglobin)	(45)
	HCT (Hematocrit)	(45)
	Platelet Count	(45)
	Retic	(45)
	Lactate	(45)
	B.S (Blood Sugar)	(45, 50)
	BUN (Blood Urea Nitrogen)	(45)
	Creatinine	(45, 50)
	Sodium	(45, 50)
	Potassium	(45, 50)
	Mg (Magnesium)	(45)
	Anion Gap	(45)
	Troponin	(45)
	Ammonia	(45)
	CK (Creatine Kinase)	(45)
	CPK (Creatine Phosphokinase)	(45, 50)
	PT, PTT (Prothrombin Time, Partial Thromboplastin Time)	(45)
	INR (International Normalized Ratio)	(45)
	ALT (Alanine Aminotransferase)	(45)
	AST (Aspartate Aminotransferase)	(45)

	**Laboratory Results**	(54, 61, 71 and 73)
	**Radiography Results**	(45, 67)
	**ECG (Electrocardiogram)**	(41, 75)
	**Test Date**	(54, 67)
Past Medical History Data	History of Addiction	(41, 45 and 68)
	History of Psychiatric Disorders	(41, 45, 50 and 68)
	Medical History	(41, 54 and 76)
	History of the Used Medications	(41, 45 and 54)
	History of Poisoning with the Toxic Substances	(45)
	Previous Suicide Attempt	(76)
	Comorbidity Diseases	(56, 67, 71, 76 and 77)
Diagnosis Data	Medical	(41)
	Psychiatric	(41)
Treatment Plan Data	**Type of Treatment**	(41, 45, 47, 50, 54, 55, 57-64, 66, 67, 69, 72, 73 and 78)
	Antidotal Therapy	(60, 64, 72 and 78)
	Antivenom Therapy	(60)
	Supportive Care—Pharmacological	(60, 72)
	SupportiveCare—Non-Pharmacological	(60, 72)
	Decontamination	(47, 60 and 78)
	Chelation Therapy	(60)
	Enhanced Elimination	(60)
	Other	(47, 64 and 72)
	**Duration of Treatment (**Days, Weeks)	(59)
	**Frequency of Treatment (**Per Day, Per Week)	(59)
	**Surgeries**	(45)
	Jejunostomy Feeding	(45)
	Laparotomy	(45)
	Gastrotomy	(45)
	Esophagectomy	(45)
	Colon Interposition	(45)
	Gastric Pull-Up	(45)
	Buginage	(45)
